# Differential Transcriptional Landscape of Vero Cells During Dengue Virus 2 Infection in the Presence of Sinococuline

**DOI:** 10.3390/microorganisms12122529

**Published:** 2024-12-08

**Authors:** Amit Garg, Rahul Shukla, Amit Kumar, Charu Aggarwal, Arnab Mukhopadhyay, Navin Khanna

**Affiliations:** 1 Translational Health, Molecular Medicine Division, International Centre for Genetic Engineering & Biotechnology, New Delhi 110067, India; 2 Division of Virus Research and Therapeutics, CSIR-Central Drug Research Institute, Lucknow 226031, India; 3 Academy of Scientific and Innovative Research, Ghaziabad 201002, India; 4 Computational Genomics Centre, Indian Council of Medical Research, New Delhi 110029, India; 5 National Institute of Immunology, New Delhi 110067, India

**Keywords:** Dengue, Sinococuline, Vero cells, transcriptome, TNF, cytokine–cytokine receptor

## Abstract

Dengue virus (DENV) is transmitted by *Aedes* genus mosquitoes and is responsible for dengue fever (DF) and other severe diseases, posing a significant challenge to the global health system. Currently, anti-dengue pharmacological treatment options are not available. Earlier, we demonstrated that Sinococuline has potent anti-dengue activity and prevents virus infection. In this study, we profile the host transcriptome response in the Vero cells after infection with DENV2 in the presence of Sinococuline, using bioinformatics to identify significant differentially expressed genes (DEGs). A total of 1510 DEGs were noted by transcriptional analysis of Vero cells that were infected with dengue virus as compared to the uninfected cells, among which 697 were upregulated and 813 were downregulated. Also, 184 out of 697 and 254 out of 817 genes were altered in dengue-infected Vero cells in the presence of Sinococuline. We found that TNF, cytokine–cytokine receptor interactions, and NF-kB signaling pathways were differentially expressed in DENV2-infected Vero cells, which was prevented by Sinococuline. The findings of this study add to our knowledge of Sinococuline’s antiviral activity in DENV2-infected Vero cells at the transcriptome level. These findings also identify potential candidate antiviral genes that can be verified for their function in the future.

## 1. Introduction

Dengue is among the most rapidly spreading diseases transmitted by arthropods, with the number of symptomatic cases doubling every ten years [1]. The disease is caused by the dengue virus (DENV), which belongs to the *Flaviviridae* family and is responsible for dengue fever [2]. DENV harbors a single-stranded, positive-sense RNA genome and is categorized into four distinct serotypes, labeled DENV1 through DENV4 [3]. However, infection with the DENV2 serotype is the most common, affecting millions per annum [1]. Presently, dengue infection is considered the most widespread arboviral disease globally, impacting public health through high rates of illness, death, and economic costs [4]. In order to reduce mortality rates, medical professionals’ primary goal is to diagnose and treat dengue quickly. Despite the urgent need for effective treatments, no specific antiviral drugs have been discovered for dengue infection. Current management focuses on supportive care, particularly fluid replacement [5]. Previous attempts to develop treatments for dengue hemorrhagic fever (DHF) have involved structure-based and fragment-based approaches aimed at modifying existing effective antiviral agents [6,7,8,9,10]. Although many studies have led to several compounds that are effective dengue virus inhibitors, only balapiravir [11], celgosivir [12], and chloroquine [13] have been evaluated in clinical trials. However, none of these drugs achieved the desired efficacy outcomes in these trials, highlighting the ongoing need for more effective treatments for dengue fever.

Interest in plant-derived drugs has grown in recent decades promoting research involving medicinal plants. Previous studies highlighted the importance of Sinococuline, a plant alkaloid derivative with a chemical formula of C_18_H_23_NO_5_ and a molecular mass of 333.4 g/mol, obtained from various plants like *Cocculus trilobus* [14] and *Cocculus hirsutus* [15]. Sinococuline was reported to exhibit various anti-inflammatory, apoptotic (on HL60 cells) [16], and antitumor activity on p388 leukemia [17]. Recently, our group showed that Sinococuline extracted from *Cocculus hirsutus* has the potential active chemical entities out of five-Coniferyl alcohol, 20-Hydroxyecdysone, Makisterone-A, Magnoflorine, and Sinococuline [15]. It has shown anti-dengue activity and ability to prevent the infection of all four serotypes (DENV1–4) in Vero cells, as assessed using a flow cytometry-based neutralization assay (FNT) [18]. The precise mechanisms underlying DENV pathogenesis and host interaction are complicated and remain elusive, even though the dengue virus employs several strategies to circumvent the immune response, especially the innate immune system [19].

In recent years, RNA sequencing technologies have been extensively used in new gene mining, differential gene expression analysis, and gene function annotation [20,21,22,23]. It offers advantages over traditional methods in terms of accuracy, coverage, and reproducibility. Currently, the frequency of application of transcriptome sequencing has increased in the study of viral infection. This has enabled thorough analyses of host defense mechanisms and immunity-antagonizing strategies at the whole mRNA level in virus infections and created new approaches that can be applied to therapeutic and preventive interventions.

In the current study, the Vero cell line from an African green monkey kidney was selected, which is widely used for studying flavivirus replication. This cell line was infected with DENV2 to investigate the modulation of transcriptome upon Sinococuline treatment. Furthermore, to comprehend the host response and to discover the functions linked with differentially expressed genes (DEGs), Gene Ontology (GO) analysis was conducted on samples collected from Sinococuline-treated cells on the 4th day after DENV2 infection. Our results exhibited that inflammatory gene expression tends to be downregulated with Sinococuline treatment in DENV2-infected cultured Vero cells. This study sheds light on the working action of Sinococuline in dengue virus pathogenesis and helps identify potential target genes for therapeutic development.

## 2. Materials and Methods

### 2.1. Cells and Viruses

For this study, Vero cells (ATCC^®^ CCL81™) derived from the kidney epithelium of a female green monkey were sourced from ATCC. These cells were grown in Dulbecco’s Modified Eagle’s Medium (DMEM) supplemented with 10% heat-inactivated fetal bovine serum and antibiotics including penicillin and streptomycin. The cell cultures were incubated at 37 °C in an atmosphere with 10% CO_2_. The DENV2 strain S16803, provided by Dr. Aravinda de Silva from the University of North Carolina, was propagated in our laboratory for use in subsequent experiments.

### 2.2. Immunofluorescence Microscopy

Vero cells were initially infected with DENV2 at a multiplicity of infection (MOI) of 1 for 2 h. Following infection, the cells were incubated for an additional 4 days and treated with an aqueous solution of Sinococuline (5 µg/mL, CAS #109351-36-2). To prepare the samples for analysis, cells were fixed in 4% paraformaldehyde for 20 min at room temperature. After fixation, the cells were washed with 1 x phosphate-buffered saline (PBS), followed by a 15 min treatment with ice-cold methanol at 4 °C and another wash with PBS.

Subsequently, the cells were blocked with 2% polyvinylpyrrolidone (PVP, Catalog No. PVP40T-1KG) for 1 h at room temperature. After blocking, the cells were washed three times with 1 x PBST (0.1% Tween 20 in 1 x PBS). The cells were then incubated with a primary antibody (3H5, 20 µg/mL) against DENV2 for 1 h at room temperature in a 10% blocking solution. Following primary antibody incubation, the cells were washed with 1 x PBST and then incubated with a 1:300 dilution of fluorescence-conjugated secondary antibody (goat anti-mouse IgG, Catalog No. AP124R) for 30 min in a 10% blocking solution. After another wash with 1 x PBST, the cells were mounted with ProLong™ Diamond Antifade Mountant with DAPI (Invitrogen, Waltham, MA, USA, Catalog No. P36962) and examined under a fluorescence microscope (Nikon, Tokyo, Japan) at 100× magnification.

### 2.3. NS1 ELISA

The secretory NS1 antigen assay was conducted using Vero cells with slight modifications to previously described methods [24]. In this assay, viral non-structural protein 1 (NS1) secreted by DENV-infected mammalian cells was captured from the culture medium. Initially, Vero cells (5.0 × 10^4^ cells/well) were seeded in 96-well plates and then infected with DENV2 at a multiplicity of infection (MOI) of 1 for 2 h at 37 °C in a 10% CO_2_ humidified incubator. Following the infection period, the overlay medium was discarded and replaced with fresh media (1 x DMEM + 2.0% ΔFBS) containing Sinococuline at a concentration of 5 µg/mL in a total volume of 200 µL. The cells were then incubated for four days.

During the incubation period, 100 µL aliquots of the culture supernatant were collected at 24 h intervals for four days. The NS1 capture ELISA was performed using the Dengue NS1 Ag Microlisa kit (J Mitra & Co., New Delhi, India, Catalog No. IR031096) according to the manufacturer’s instructions. For the assay, 50 µL of the diluted culture supernatant (1:200) was added to microwells pre-coated with anti-Dengue NS1 antibodies, along with a working conjugate (NS1 antibodies linked to horseradish peroxidase). The mixture was incubated at 37 °C for 90 min. The microwells were then washed six times with 1x washing buffer provided with the kit, and a working substrate solution was added and incubated at room temperature for 15 min. The reaction was terminated with 1N H_2_SO_4_ and the absorbance was measured at 450 nm. Data analysis was conducted using GraphPad Prism 9 (Version 9.5.0).

### 2.4. Sample Preparation and RNA Extraction

Vero cells, cultured in T-75 flasks at a density of 2.0 × 10^6^ cells per flask, were initially infected with DENV2 strain S16803 at a multiplicity of infection (MOI) of 1 for 2 h. Following the infection period, the cells were incubated for 4 days and treated with Sinococuline at a concentration of 5 µg/mL. The cells treated with an equal volume of DMEM without Sinococuline served as the untreated control. After the incubation, the cells were washed with 1 x phosphate-buffered saline (PBS) and total RNA was extracted using TRIzol reagent (Invitrogen, Waltham, MA, USA, Catalog No. 15596-018).

### 2.5. Library Preparation and RNA Sequencing

Samples were prepared for transcriptomic sequencing with three biological replicates per group. The quantity of the extracted RNA was assessed using a Qubit 4.0 fluorometer (Thermo Fisher, Waltham, MA, USA, Catalog No. Q33238) with the RNA HS assay kit (Thermo Fisher, Waltham, MA, USA, Catalog No. Q32851) following the manufacturer’s guidelines. RNA purity was evaluated using a Nanodrop 1000 (Waltham, MA, USA) and RNA integrity was determined by analyzing the samples on a TapeStation with HS RNA ScreenTape (Agilent, Santa Clara, CA, USA).

The cDNA library was constructed using the TruSeq^®^ Stranded Total RNA kit. The final libraries were quantified using the Qubit 4.0 fluorometer (Thermo Fisher, Catalog No. Q33238) with the DNA HS assay kit (Thermo Fisher, Catalog No. Q32851). Library insert sizes were analyzed on a TapeStation 4150 (Agilent) with D1000 ScreenTape (Agilent, Catalog No. 5067-5582) following the manufacturer’s protocols. Sequencing was conducted using an Illumina NovaSeq 6000 sequencer (Illumina, San Diego, CA, USA).

### 2.6. Sequencing Analysis

Quality control of the raw sequencing reads was conducted using FastQC version 0.11.9, Trimmomatic version 0.39, and MultiQC version 1.12. Adapter sequences were removed and only high-quality reads, defined as having an average base call quality score of 30 or greater, were retained. On average, each sample yielded 35.66 million filtered clean reads, with an average data size of 12 GB, which was stored in FASTQ format.

The reference genome of the *Chlorocebus sabaeus* sequence was indexed with HISAT2 (Hierarchical Indexing for Spliced Alignment of Transcripts 2). The raw reads were then aligned to this indexed reference using splice site information from the genome annotation. The resulting BAM files were processed using featureCounts [25]. Differentially expressed genes (DEGs) were identified using criteria of *p* < 0.05 and fold change (FC) ≥ 1.5. Gene Ontology (GO) and pathway enrichment analyses were performed using the DAVID tool (v6.8).

### 2.7. Gene Expression Analysis

For the quantitative assessment of gene expression, clean reads were aligned to the reference gene sequences using HISAT2 [26]. Gene expression levels in each sample were determined with DESeq2, utilizing the default settings [27]. Differentially expressed genes (DEGs) were identified based on criteria of fold change (FC) ≥ 1.5 and a *p*-value ≤ 0.05.

Gene Ontology (GO) term and pathway enrichment analyses for the DEGs were performed using the DAVID database [28]. GO terms and pathways with a false discovery rate (FDR) of ≤0.1 were considered significantly enriched among the candidate genes. The results of the GO and pathway enrichment analyses were visualized using Hiplot [29].

## 3. Results

### 3.1. Infection of Vero Cells with DENV2

**Figure 1:**
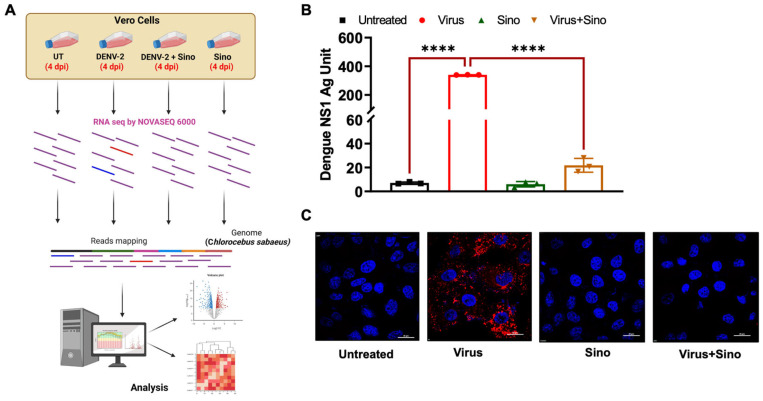
Workflow and confirmation of protective function of Sinococuline in DENV2-infected cultured Vero cells. (**A**) Workflow: Vero cells were infected with DENV2 (MOI 1) and treated with Sinococuline and vehicle control, followed by sample collection at 4 dpi. Samples from each group were collected in triplicate. Total RNA was extracted, and RNA sequencing was performed. (**B**) Estimation of NS1 levels in Sinococuline- and vehicle control-treated DENV2-infected Vero cells. The supernatant was collected at 4 dpi and NS1 Microlisa was performed to quantify the NS1 antigen levels as per the manufacturer’s instructions. Data represent the means ± SD. Statistically significant differences between individual groups were determined by using one-way ANOVA, **** *p ≤* 0.0001. (**C**) Immunofluorescence staining of the DENV2 infection in the Vero cells: 4 dpi cells were fixed and DENV2 (red) presence was detected in the Vero cells using anti-DENV2 EDIII mAb, 3H5. Nuclei (blue) were shown by 4′,6′-diamidino-2-phenylindole (DAPI) staining. The images of cells were acquired by confocal microscope (Nikon) at a 100× magnification. DENV2: dengue virus 2; MOI: multiplicity of infection; dpi: days post-infection; NS1: non-structural protein 1; Sino: Sinococuline; and UT: untreated.

To gain insight into the dynamics of the host transcriptional response of drug therapy in DENV2 infection during the late stage, cultured Vero cells were infected with DENV2 strain S16803 at MOI 1 for 4 days and Sinococuline, an anti-dengue drug candidate [15], was added to the infected cells (Figure 1A). The concentration of 5 µg/mL of Sinococuline was chosen, which is below the previously determined CC_50_ [30]. Here, we refer to the vehicle-treated group as the virus-infected group and the Sinococuline-treated, DENV2-infected Vero cells as Sinococuline-treated cells.

To validate Sinococuline antiviral activity, supernatant of cultured Vero cells infected with DENV2 were harvested at 0, 1, 2, 3, and 4 dpi (days post-infection) and non-structural 1 protein (NS1) levels at each time point were measured using ELISA (Supplementary Materials Figure S1A). The results indicated that NS1 antigen levels gradually increased up to day 4 in vehicle-treated or virus-infected cells, while this increase was not noticed in Sinococuline-treated cells as observed previously [30]. DENV2 replication was successfully inhibited after Sinococuline treatment as assessed by NS1 antigen levels at 4 days post-infection (Figure 1B). In addition to detecting secreted NS1 levels, we also looked for the presence of intracellular viruses in the cultured Vero cells after vehicle and Sinococuline treatment at 4 dpi. No infectious virus particles were detected intracellularly after Sinococuline treatment, in contrast to the vehicle-treated Vero cells (Figure 1C). In both assays, the S16803 strain proliferated efficiently without Sinococuline (Figure 1B,C). Therefore, untreated, vehicle-treated, Sinococuline-treated uninfected and Sinococuline-treated DENV2-infected Vero cells were harvested at 4 dpi for RNA sequencing analysis. These results indicated that Sinococuline effectively prevents the DENV2 strain S16803 replication in the cultured Vero cells.

### 3.2. RNA-Seq Data and DEG Analysis

To investigate each group’s reproducibility and variation, principal component analysis (PCA) was performed using the gene expression levels. Every sample with the same gene expression profile was clustered in proximity to each other, indicating that each treatment’s repeatability was satisfactory and the specificity between groups was apparent and suitable for further application (Supplementary Materials Figure S1B).

**Figure 2:**
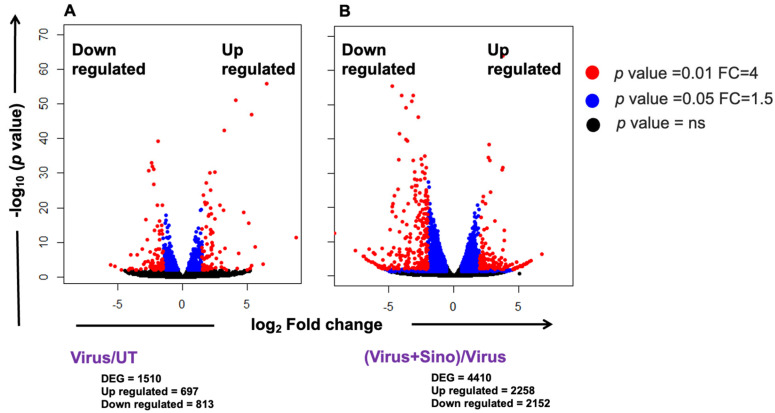
Volcano plot of differentially expressed genes (DEGs). (**A**) DEGs of the virus-infected group as compared to the mock-infected or untreated group. The panel’s left side shows 813 downregulated DEGs and the right side shows 697 upregulated DEGs. (**B**) DEGs of the Virus + Sino-treated group as compared to the virus-infected group. The left side of the panel shows 2152 downregulated DEGs and the right side shows 2258 upregulated DEGs. The red dot represents a 4-fold change (FC) and the *p*-value is 0.01; the blue dot represents a 1.5-fold change and the *p*-value is 0.05; and the black dot represents the non-significant *p*-value. Sino: Sinococuline; FC: fold change.

In order to visualize the transcriptome changes induced after DENV2 infection and Sinococuline treatment in the infected Vero cells at 4 dpi, a total of 12 sequencing libraries were prepared and subjected to sequencing. Three biological replicates were present in each group. The virus-infected group included 37,214,970, 57,831,192, and 42,330,298; the Sinococuline-treated group had 49,091,224, 42,764,790, and 40,573,826; the untreated group included 48,735,884, 56,685,162, and 51,483,910; and the Sinococuline-treated uninfected group had 46,081,300, 22,524,536, and 48,541,120 raw reads. After removing the adapter and low-quality reads, there were 32,078,382, 41,547,576, and 32,136,172 clean reads in the infected group; 33,382,600, 34,665,906, and 33,333,048 clean reads in the Sinococuline-treated group; 30,960,596, 44,291,342, and 38,571,058 clean reads in the untreated group; and 34,467,922, 18,014,848, and 35,300,050 clean reads in the Sinococuline-treated uninfected group. Differentially expressed genes (DEGs) were noted between the infected and Sinococuline-treated groups using the DESeq2 R package and are shown in volcano plots (Figure 2). A *p* < 0.05 and fold change (FC) ≥ 1.5 were considered as the thresholds. The results showed that 1510 and 4410 DEGs were identified that may be linked with DENV2 infection when compared to the untreated group (Figure 2A) and associated with Sinococuline treatment when compared to the virus-infected group, respectively (Figure 2B) (Supplementary Materials Tables S1 and S2). Among them, 697 genes in DENV2 infection and 2258 genes in Sinococuline treatment were upregulated. In addition, 813 genes in the infection group and 2152 genes in the Sinococuline treatment group were downregulated. Sinococuline treatment in uninfected cells led to 4896 DEGs of which 2318 were upregulated and 2578 were downregulated when compared to the untreated group (Supplementary Materials Figure S2 and Table S3). Altogether, these data indicated that the infection of cultured Vero cells by DENV2 strain S16803 and Sinococuline treatment in the infection condition elicits broad-range alterations in the gene expression patterns of the cells.

### 3.3. Comparison Between DENV2-Infected and Sinococuline-Treated Infected Group Transcriptomics

**Figure 3:**
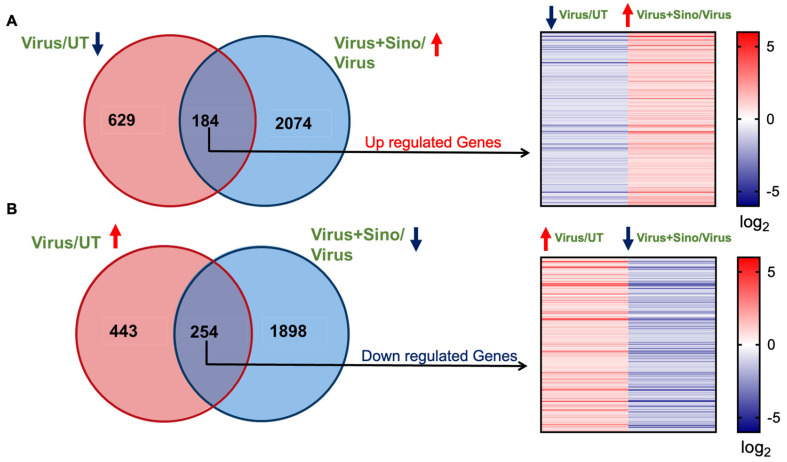
Venn diagrams illustrate the number of genes uniquely expressed under each condition, with overlapping areas indicating genes expressed in both conditions. (**A**) 184 DEGs were upregulated in the Virus + Sino group compared to the virus group. A heat map shows the range of log2 fold changes in the genes. (**B**) 254 DEGs were shared between the downregulated Virus + Sino group and upregulated virus groups. The heat map shows the range of log2 fold changes in the genes. Sino: Sinococuline; DEGs: differentially expressed genes.

To compare the transcriptomic profile of the DENV2-infected and Sinococuline-treated groups, Venn diagrams were constructed with the list of DEGs, showing the unique and overlapping genes between the data sets. To this end, of the set of DEGs that were observed to be changed after 4 dpi, 184 genes were upregulated with Sinococuline treatment that was downregulated in virus-infected groups, and their log_2_ fold changes are presented using a heat map (Figure 3A and Supplementary Materials Table S4). Moreover, 254 DEGs were suppressed in Sinococuline treatment that were upregulated in DENV2 infection at 4 dpi and their log_2_ fold changes are shown in the heat map (Figure 3B and Supplementary Materials Table S5). However, treatment with Sinococuline alone majorly alters a different set of genes as compared to Sinococuline treatment in DENV2-infected Vero cells (Supplementary Materials Figure S3). Overall, these results indicated that a total of 438 DEGs were directly altered in the Sinococuline treatment in infected Vero cells.

### 3.4. GO Enrichment Analysis of DEGs

**Figure 4:**
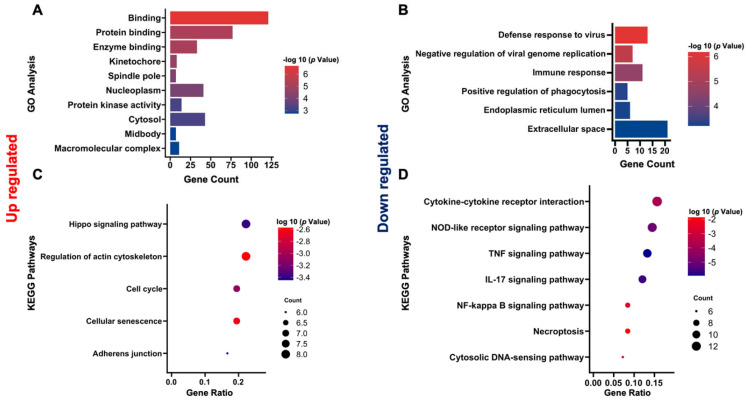
GO enrichment and KEGG pathway analysis. The bar graph represents the most significantly enriched GO terms for the (**A**) upregulated DEGs and (**B**) downregulated DEGs. The bubble plot displays the KEGG pathways with the highest levels of enrichment for the (**C**) upregulated and (**D**) downregulated DEGs. The color represents the *p*-value of the enrichment. The size of the bubble represents the gene ratio. DEGs: differentially expressed genes; GO: Gene Ontology; and KEGG: Kyoto Encyclopedia of Genes and Genomics.

To further comprehend the relevant functions of unique and overlapping DEGs, Gene Ontology enrichment analysis (GO) was performed. In GO analysis of 184 genes that were shared between the Sinococuline-treated (upregulated) and virus-infected (downregulated) groups, 72 terms were obtained which comprised 36 biological processes (BPs), 23 cellular components (CCs), and 13 molecular functions (MFs) terms. Among the CC terms, the upregulated DEGs mainly included macromolecular complex kinetochore, spindle pole, and nucleoplasm cytosol; and in MF terms, the most enriched categories were enzyme binding, protein binding, and protein kinase activity (Figure 4A). Among the 254 downregulated DEGs, 66 terms were found, comprising 49 BP, 4 CC, and 13 MF terms. Regarding the BP category, downregulated DEGs were involved in the negative regulation of viral genome replication, positive regulation of phagocytosis immune response, and defense response to the virus. Among the CC terms, the DEGs were enriched in categories that were relevant to the endoplasmic reticulum lumen and extracellular space (Figure 4B).

### 3.5. KEGG Enrichment Analysis of DEGs

For ease of understanding, all selected DEGs were functionally categorized according to the Kyoto Encyclopedia of Genes and Genomics (KEGG) pathway analysis. Twelve KEGG pathways were enriched in the upregulated DEGs. Subsequently, these pathways were filtered using thresholds of *p*-value < 0.05 and a false discovery rate (FDR) < 0.1. Under these criteria, the highly abundant pathways included the Hippo signaling pathway, regulation of actin cytoskeleton, cell cycle, cellular senescence, and adherens junction (Figure 4C). The downregulated DEGs were distributed in a total of 36 pathways. After filtering out from the cutoff, the highly enriched pathways were comprised of cytokine–cytokine receptor interaction, tumor necrosis factor (TNF) signaling, Interleukin-17 (IL-17) signaling, and nuclear factor kappa B (NF-kappa B) signaling pathways (Figure 4D). These results indicated that Sinococuline treatment in infected Vero cells regulated multiple signaling cascades and reduced the intense inflammatory response.

**Figure 5:**
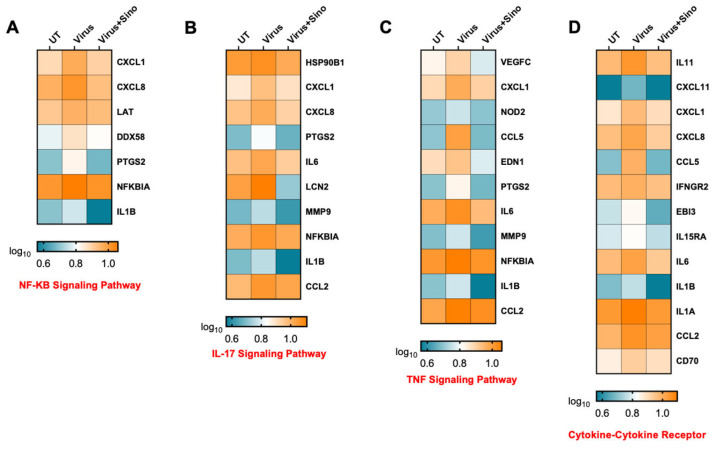
Heat map illustrating gene expression levels across four KEGG pathways: (**A**) NF-kB signaling pathway, (**B**) IL-17 signaling pathway, (**C**) TNF signaling pathway, and (**D**) cytokine–cytokine receptor signaling pathway. KEGG: Kyoto Encyclopedia of Genes and Genomics; NF-kB: nuclear factor kappa B; IL-17: Interleukin-17; and TNF: tumor necrosis factor.

To analyze the host response during the Sinococuline treatment in DENV2 infection, heatmaps were generated to display the gene expression trend in NF-kB signaling pathway, IL-17, TNF signaling pathways, and the cytokine–cytokine receptor pathway (Figure 5A–D). The TNF signaling cascade consists of CXCL1, CCL2, CCL5, IL6, and IL1B genes and cytokine–cytokine receptor signaling cascade-related genes (CXCL8, IL11, IFNGR2, IL1A, and IL15RA). These results revealed sets of genes that were downregulated in Sinococuline treatment in DENV2-infected Vero cells at 4 dpi and suggested that Sinococuline might modulate the inflammatory genes in DENV2 infection.

## 4. Discussion

Dengue virus can cause illnesses from classical dengue fever (DF) to life-threatening ones like dengue hemorrhagic fever (DHF) and dengue shock syndrome (DSS) [31]. Humans are the sole hosts for epidemic DENV strains, which have evolved complex mechanisms to antagonize innate immune responses [32]. Dengue has imposed a significant burden on the global healthcare system. Despite our extensive knowledge of dengue pathogenesis, the activation of many signaling cascades and the transcriptional factors (TFs) that contribute to the diverse disease symptoms in the host remains elusive. In an effort to better understand the underlying biology of the host response to DENV2 infection in the presence of Sinococuline treatment and identify the potential therapeutic genes, we used RNA-Seq technology to visualize the transcriptional response in the host Vero cells in both conditions. In this study, samples were collected at 4 dpi that exhibited high levels of secretory NS1 and virus particles upon DENV2 infection in cultured Vero cells for transcriptome sequencing. Data presented in this study confirm our previous finding that secretory NS1 levels and virus titer increased up to days 3 to 4 and attained a plateau [24]. To avoid cytopathic progression that may affect the quality of RNA, we focused on day 4 post-infection to identify key transcriptomic alterations in host cells in response to DENV2 infection and evaluate the impact of the treatment.

A total of 1510 DEGs were identified in the DENV2-infected group as compared to the untreated group, among which 697 genes were activated, and 813 genes were suppressed. Interestingly, Sinococuline treatment led to the upregulation of 184 genes out of 813 DEGs that were suppressed in the virus infection condition. And 254 genes were downregulated after the treatment, out of 697 activated DEGs of virus infection in the host cell condition. Further analysis included enrichment studies to explore the functions of differentially expressed genes (DEGs) affected by Sinococuline treatment. The enrichment results highlighted that the most downregulated DEGs were associated with inflammatory response pathways, including IL-17 signaling, NF-kB signaling, and TNF signaling cascades. Earlier, our group reported the pan anti-dengue activity of *Cocculus hirsutus* (AQCH), a phytopharmaceutical [15], where we observed that AQCH extract (25 mg/kg body weight) provided protection from dengue severity in a primary and secondary dengue murine model, AG129 mice, when administered three or four times a day. Dengue progression led to various detrimental outcomes such as plasma leakage, hemorrhage, and thrombocytopenia. AQCH prevented the development of these detrimental outcomes in dengue-infected AG129 mice [15]. Moreover, elevated levels of MMP-9 (matrix metalloproteinase-9) observed in the sera of DHF patients contribute significantly to the breakdown of adhesion and tight junction proteins, which leads to hyperpermeability and vascular leakage in human endothelial cells and murine tissues. [33]. Interestingly, Sinococuline inhibited the secreted NS-1 levels (Figure 1B), which may lead to a reduction in the recruitment of MMP-9 and prevent the mice from developing vascular leakage [30].

Moreover, TNF signaling [34], IL-17 signaling [35], and NF-kB signaling [36,37] cascades play key roles in the progression of dengue severity. Many inhibitors were designed to block these pathways to prevent the progression of complications such as DHF/DSS [34]. However, the precise mechanisms of DHF/DSS have not been elucidated. It is believed that several proinflammatory mediators, such as nitric oxide (NO) and tumor necrosis factor-*α* (TNF-*α*), are involved in hemorrhage development [38,39]. Sinococuline treatment blocked dengue virus replication (Figure 1C), which resulted in the downregulation of TNF and other proinflammatory cascades. The in vivo study from our group highlighted that Sinococuline treatment in dengue-infected mice reduced the viral loads in the vital organs as well as tissue proinflammatory cytokine levels like TNF-α and IL-6 [30]. Previous research has demonstrated that TNF-α has a significant contribution to the development of vascular leakage by stimulating reactive oxygen and nitrogen intermediates [40,41]. Sinococuline treatment effectively lowered the TNF and RNI (reactive nitrogen intermediate) levels in LPS (lipid polysaccharides)-induced rat alveolar macrophages [16]. Therefore, the decrease in vascular leakage observed with Sinococuline treatment may be due to the reduction in TNF and RNI levels.

Beyond their essential role in immune system development and homeostasis, cytokines and chemokines also contribute to either beneficial or harmful immune and inflammatory responses. During infection and inflammation, these molecules are actively secreted and relay signals to cells through their specific receptors. Investigating the cytokine–cytokine receptor interactions within the KEGG pathways, we observed notable alterations in the expression of several key cytokines and their receptors. CCL2, CCL5, CXCL11, CXCL1, CXCL8, IL-11, IL-1A and B, and IL-6 were noticed to be downregulated in response to Sinococuline treatment in DENV infection. Moreover, IL-6 was also downregulated, which was increased in other flavivirus infections like Zika [42], West Nile virus (WNV) [43], and Japanese encephalitis virus (JEV) [44].

Our study has several limitations. We used an in vitro model of dengue virus infections to identify potential protective pathways regulated by Sinococuline treatment. However, this model may not accurately represent the effectiveness of Sinococuline in dengue patients. Our experiments were conducted using Vero cells, as recommended by WHO guidelines. However, this limitation may restrict our understanding of the diverse interactions between the dengue virus and various cell types. Therefore, additional studies are necessary to determine Sinococuline’s anti-dengue potential for treating dengue illness due to the lack of an ideal model system.

In summary, we extensively investigated how the host response is orchestrated at the transcriptional level in the later phase of Sinococuline treatment in DENV2 infection. We identified a significant number of regulatory and potential antiviral genes associated with the virus, offering foundational insights and guidance for future research on host–pathogen interactions. The analysis revealed that differentially expressed genes primarily involved in cellular transcription, inflammatory responses, and signal transduction were central to the host’s reaction to DENV2 infection. Notably, signaling pathways such as the TNF pathway and cytokine–cytokine receptor interactions, which are crucial in host–virus dynamics, were downregulated following Sinococuline treatment. This study highlights the protective effects of Sinococuline during DENV pathogenesis and provides a valuable resource for future research on dengue. Understanding these genes and pathways is crucial for developing new strategies to target viral replication. Additionally, our findings suggest potential new approaches for patient protection, especially in regions where dengue is endemic.

## Data Availability

All data generated or analyzed during this study are included in this article (and its Supplementary Materials). The sequencing data are available as BioProject ID: PRJEB65277; IBDC study accession: INRP000086.

## Supplementary Materials

The following supporting information can be downloaded at: https://www.mdpi.com/article/10.3390/microorganisms12122529/s1, Figure S1: NS-1 ELISA and basic information of RNA-seq results; Table S1: (related to Figure 2A) DEGs in Virus-infected group; Table S2: (related to Figure 2B) DEGs in Sinococuline-treated group; Table S3: (related to Supplementary Figure S2) DEGs in Sinococuline alone-treated group; Table S4: (related to Figure 3A) Log2 fold change of virus-infected downregulated and Sinococuline-treated upregulated DEGs; Table S5: (related to Figure 3B) Log2 fold change of virus-infected upregulated and Sinococuline-treated downregulated DEGs.

## References

[1] Stanaway J.D., Shepard D.S., Undurraga E.A., Halasa Y.A., Coffeng L.E., Brady O.J., Hay S.I., Bedi N., Bensenor I.M., Castaneda-Orjuela C.A. (2016). The global burden of dengue: An analysis from the Global Burden of Disease Study 2013. Lancet Infect. Dis..

[2] Back A.T., Lundkvist A. (2013). Dengue viruses—An overview. Infect. Ecol. Epidemiol..

[3] Katzelnick L.C., Fonville J.M., Gromowski G.D., Bustos Arriaga J., Green A., James S.L., Lau L., Montoya M., Wang C., VanBlargan L.A. (2015). Dengue viruses cluster antigenically but not as discrete serotypes. Science.

[4] Sabir M.J., Al-Saud N.B.S., Hassan S.M. (2021). Dengue and human health: A global scenario of its occurrence, diagnosis and therapeutics. Saudi J. Biol. Sci..

[5] (2009). Dengue: Guidelines for Diagnosis, Treatment, Prevention and Control.

[6] Jadhav M.P. (2014). High-throughput screening (HTS) for the identification of novel antiviral scaffolds. Clin. Pharmacol. Drug Dev..

[7] Smith T.M., Lim S.P., Yue K., Busby S.A., Arora R., Seh C.C., Wright S.K., Nutiu R., Niyomrattanakit P., Wan K.F. (2015). Identifying initiation and elongation inhibitors of dengue virus RNA polymerase in a high-throughput lead-finding campaign. J. Biomol. Screen..

[8] Yokokawa F., Nilar S., Noble C.G., Lim S.P., Rao R., Tania S., Wang G., Lee G., Hunziker J., Karuna R. (2016). Discovery of Potent Non-Nucleoside Inhibitors of Dengue Viral RNA-Dependent RNA Polymerase from a Fragment Hit Using Structure-Based Drug Design. J. Med. Chem..

[9] Anusuya S., Velmurugan D., Gromiha M.M. (2016). Identification of dengue viral RNA-dependent RNA polymerase inhibitor using computational fragment-based approaches and molecular dynamics study. J. Biomol. Struct. Dyn..

[10] Anusuya S., Gromiha M.M. (2017). Quercetin derivatives as non-nucleoside inhibitors for dengue polymerase: Molecular docking, molecular dynamics simulation, and binding free energy calculation. J. Biomol. Struct. Dyn..

[11] Nguyen N.M., Tran C.N., Phung L.K., Duong K.T., Huynh Hle A., Farrar J., Nguyen Q.T., Tran H.T., Nguyen C.V., Merson L. (2013). A randomized, double-blind placebo controlled trial of balapiravir, a polymerase inhibitor, in adult dengue patients. J. Infect. Dis..

[12] Low J.G., Sung C., Wijaya L., Wei Y., Rathore A.P.S., Watanabe S., Tan B.H., Toh L., Chua L.T., Hou Y. (2014). Efficacy and safety of celgosivir in patients with dengue fever (CELADEN): A phase 1b, randomised, double-blind, placebo-controlled, proof-of-concept trial. Lancet Infect. Dis..

[13] Tricou V., Minh N.N., Van T.P., Lee S.J., Farrar J., Wills B., Tran H.T., Simmons C.P. (2010). A randomized controlled trial of chloroquine for the treatment of dengue in Vietnamese adults. PLoS Negl. Trop. Dis..

[14] Hitotsuyanagi Y., Ikuta H., Nishimura K., Takeya K., Itokawa H. (1994). Synthesis of an Antitumour Alkaloid Sinococuline from Sinomenine. J. Chem. Soc. Chem. Commun..

[15] Shukla R., Rajpoot R.K., Poddar A., Ahuja R., Beesetti H., Shanmugam R.K., Chaturvedi S., Nayyar K., Singh D., Singamaneni V. (2021). Cocculus hirsutus-Derived Phytopharmaceutical Drug Has Potent Anti-dengue Activity. Front. Microbiol..

[16] Liu W.K., Wang X.K., Che C.T. (1996). Cytotoxic effects of sinococuline. Cancer Lett..

[17] Itokawa H., Tsuruoka S., Takeya K., Mori N., Sonobe T., Kosemura S., Hamanaka T. (1987). An antitumor morphinane alkaloid, sinococuline, from Cocculus trilobus. Chem. Pharm. Bull..

[18] Kraus A.A., Messer W., Haymore L.B., de Silva A.M. (2007). Comparison of plaque- and flow cytometry-based methods for measuring dengue virus neutralization. J. Clin. Microbiol..

[19] Kao Y.T., Lai M.M.C., Yu C.Y. (2018). How Dengue Virus Circumvents Innate Immunity. Front. Immunol..

[20] Greenbaum D., Colangelo C., Williams K., Gerstein M. (2003). Comparing protein abundance and mRNA expression levels on a genomic scale. Genome Biol..

[21] Marcotte E.M., Pellegrini M., Thompson M.J., Yeates T.O., Eisenberg D. (1999). A combined algorithm for genome-wide prediction of protein function. Nature.

[22] Morin R., Bainbridge M., Fejes A., Hirst M., Krzywinski M., Pugh T., McDonald H., Varhol R., Jones S., Marra M. (2008). Profiling the HeLa S3 transcriptome using randomly primed cDNA and massively parallel short-read sequencing. Biotechniques.

[23] Byrne A., Cole C., Volden R., Vollmers C. (2019). Realizing the potential of full-length transcriptome sequencing. Philos. Trans. R. Soc. Lond. B Biol. Sci..

[24] Sood R., Raut R., Tyagi P., Pareek P.K., Barman T.K., Singhal S., Shirumalla R.K., Kanoje V., Subbarayan R., Rajerethinam R. (2015). Cissampelos pareira Linn: Natural Source of Potent Antiviral Activity against All Four Dengue Virus Serotypes. PLoS Negl. Trop. Dis..

[25] Liao Y., Smyth G.K., Shi W. (2014). featureCounts: An efficient general purpose program for assigning sequence reads to genomic features. Bioinformatics.

[26] Kim D., Langmead B., Salzberg S.L. (2015). HISAT: A fast spliced aligner with low memory requirements. Nat. Methods.

[27] Love M.I., Huber W., Anders S. (2014). Moderated estimation of fold change and dispersion for RNA-seq data with DESeq2. Genome Biol..

[28] Sherman B.T., Hao M., Qiu J., Jiao X., Baseler M.W., Lane H.C., Imamichi T., Chang W. (2022). DAVID: A web server for functional enrichment analysis and functional annotation of gene lists (2021 update). Nucleic Acids Res..

[29] Li J., Miao B., Wang S., Dong W., Xu H., Si C., Wang W., Duan S., Lou J., Bao Z. (2022). Hiplot: A comprehensive and easy-to-use web service for boosting publication-ready biomedical data visualization. Brief. Bioinform..

[30] Shukla R., Ahuja R., Beesetti H., Garg A., Aggarwal C., Chaturvedi S., Nayyar K., Arora U., Lal A.A., Khanna N. (2023). Sinococuline, a bioactive compound of Cocculus hirsutus has potent anti-dengue activity. Sci. Rep..

[31] Khetarpal N., Khanna I. (2016). Dengue Fever: Causes, Complications, and Vaccine Strategies. J. Immunol. Res..

[32] Green A.M., Beatty P.R., Hadjilaou A., Harris E. (2014). Innate immunity to dengue virus infection and subversion of antiviral responses. J. Mol. Biol..

[33] Pan P., Li G., Shen M., Yu Z., Ge W., Lao Z., Fan Y., Chen K., Ding Z., Wang W. (2021). DENV NS1 and MMP-9 cooperate to induce vascular leakage by altering endothelial cell adhesion and tight junction. PLoS Pathog..

[34] Watanabe S., Chan K.W., Wang J., Rivino L., Lok S.M., Vasudevan S.G. (2015). Dengue Virus Infection with Highly Neutralizing Levels of Cross-Reactive Antibodies Causes Acute Lethal Small Intestinal Pathology without a High Level of Viremia in Mice. J. Virol..

[35] Jain A., Pandey N., Garg R.K., Kumar R. (2013). IL-17 level in patients with Dengue virus infection & its association with severity of illness. J. Clin. Immunol..

[36] Jan J.T., Chen B.H., Ma S.H., Liu C.I., Tsai H.P., Wu H.C., Jiang S.Y., Yang K.D., Shaio M.F. (2000). Potential dengue virus-triggered apoptotic pathway in human neuroblastoma cells: Arachidonic acid, superoxide anion, and NF-kappaB are sequentially involved. J. Virol..

[37] Marianneau P., Cardona A., Edelman L., Deubel V., Despres P. (1997). Dengue virus replication in human hepatoma cells activates NF-kappaB which in turn induces apoptotic cell death. J. Virol..

[38] Martina B.E., Koraka P., Osterhaus A.D. (2009). Dengue virus pathogenesis: An integrated view. Clin. Microbiol. Rev..

[39] Fernandez-Mestre M.T., Gendzekhadze K., Rivas-Vetencourt P., Layrisse Z. (2004). TNF-alpha-308A allele, a possible severity risk factor of hemorrhagic manifestation in dengue fever patients. Tissue Antigens.

[40] Meena A.A., Murugesan A., Sopnajothi S., Yong Y.K., Ganesh P.S., Vimali I.J., Vignesh R., Elanchezhiyan M., Kannan M., Dash A.P. (2020). Increase of Plasma TNF-alpha Is Associated with Decreased Levels of Blood Platelets in Clinical Dengue Infection. Viral Immunol..

[41] Yen Y.T., Chen H.C., Lin Y.D., Shieh C.C., Wu-Hsieh B.A. (2008). Enhancement by tumor necrosis factor alpha of dengue virus-induced endothelial cell production of reactive nitrogen and oxygen species is key to hemorrhage development. J. Virol..

[42] Magoro T., Dandekar A., Jennelle L.T., Bajaj R., Lipkowitz G., Angelucci A.R., Bessong P.O., Hahn Y.S. (2019). IL-1beta/TNF-alpha/IL-6 inflammatory cytokines promote STAT1-dependent induction of CH25H in Zika virus-infected human macrophages. J. Biol. Chem..

[43] Zidovec-Lepej S., Vilibic-Cavlek T., Barbic L., Ilic M., Savic V., Tabain I., Ferenc T., Grgic I., Gorenec L., Bogdanic M. (2021). Antiviral Cytokine Response in Neuroinvasive and Non-Neuroinvasive West Nile Virus Infection. Viruses.

[44] Zhou Y., Bian P., Du H., Wang T., Li M., Hu H., Ye C., Zheng X., Zhang Y., Lei Y. (2022). The Comparison of Inflammatory Cytokines (IL-6 and IL-18) and Immune Cells in Japanese Encephalitis Patients with Different Progression. Front. Cell. Infect. Microbiol..

