# Supplementary material for: Differential Transcriptional Landscape of Vero Cells During Dengue Virus 2 Infection in the Presence of Sinococuline
Source: Microorganisms. 2024 Dec 8;12(12):2529. doi: 10.3390/microorganisms12122529 (PMC11678108; doi:10.3390/microorganisms12122529)
Supplement: Supplementary file 1 [file microorganisms-12-02529-s001.zip › microorganisms-3228399-supplementary.pdf]

Differential Transcriptional Landscape of Vero Cells During Dengue Virus 2 Infection in the Presence of Sinococuline

Amit Garg [1,*], Rahul Shukla [2,3], Amit Kumar [4], Charu Aggarwal [1], Arnab Mukhopadhyay [5] and Navin Khanna [1,*]

1 Translational Health, Molecular Medicine Division, International Centre for Genetic Engineering & Biotechnology, New Delhi 110067, India

2 Division of Virus Research and Therapeutics, CSIR-Central Drug Research Institute, Lucknow 226031, India

3 Academy of Scientific and Innovative Research, Ghaziabad 201002, India

4 Computational Genomics Centre, Indian Council of Medical Research, New Delhi 110029, India

5 National Institute of Immunology, New Delhi 110067, India

* Correspondence: amith.garg@gmail.com (A.G.); navinkhanna5@gmail.com (N.K.)

Supplementary Figures

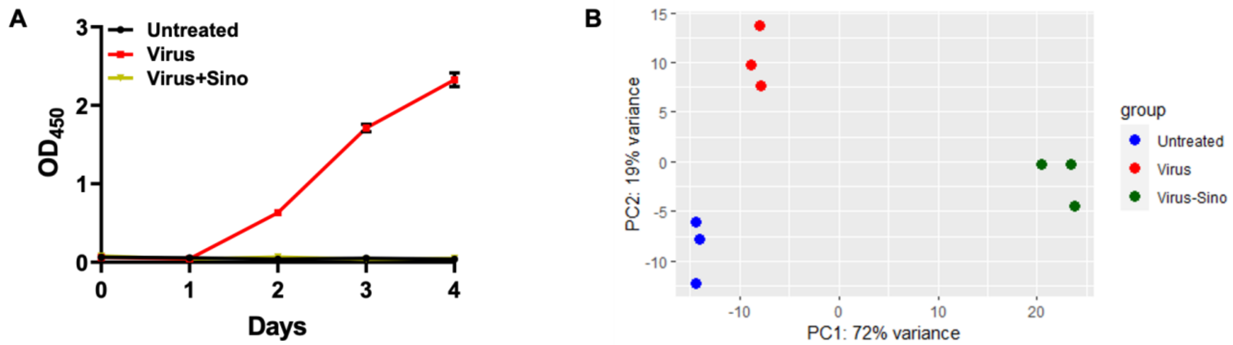

Supplementary Figure S1. NS-1 ELISA and basic information of RNA-seq results. (A) ELISA shows the secretory NS-1 levels from day 1–4 DENV2 infected cells and after treatment with Sinococuline (B) Dot plot shows the principal component analysis (PCA) of each sample. Sino: Sinococuline.

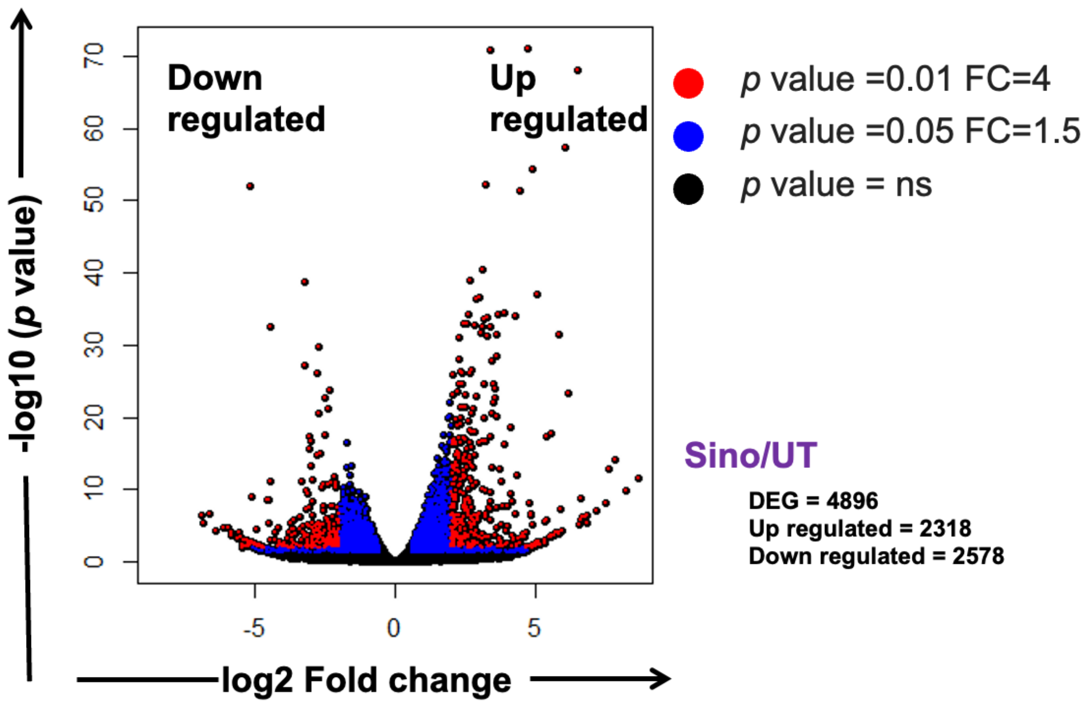

Supplementary Figure S2. Volcano plot of differentially expressed genes (DEGs). DEGs of the Sinococuline alone treated as compared to the untreated group. The panel's left side shows 2578 downregulated DEGs, and the right side shows 2318 upregulated DEGs.

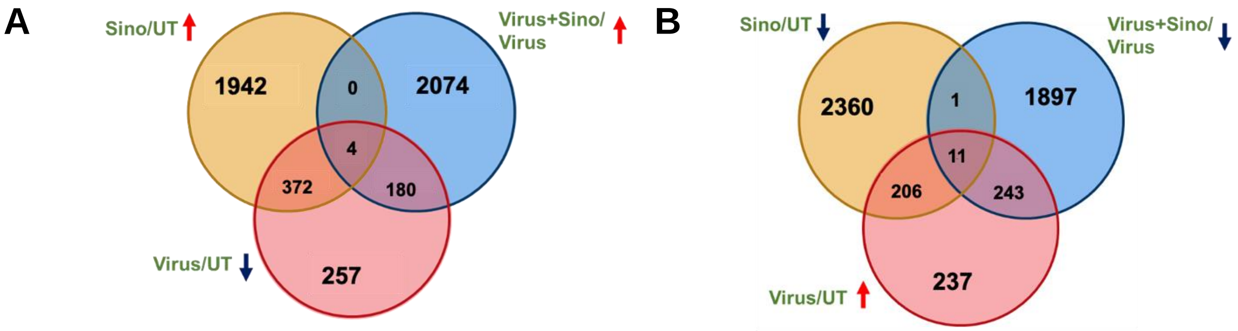

Supplementary Figure S3. Venn diagrams illustrate the number of genes uniquely expressed under each condition, with overlapping areas indicating genes expressed in all three conditions. (A) 4 DEGs were upregulated in the Virus + Sino group and Sino alone compared to the virus group (B) 11 DEGs were shared between downregulated Virus + Sino, Sino alone groups and upregulated virus groups. Sino: Sinococuline, DEGs: Differentially expressed genes.
